# Inferior Vena Cava Catheterization During Percutaneous Nephrolithotomy

**DOI:** 10.1002/ccr3.70748

**Published:** 2025-09-04

**Authors:** Dor Golomb, Michael Doberman, Sergey Litvin, Yuval Avda, Amir Cooper, Orit Raz

**Affiliations:** ^1^ Department of Urology Samson Assuta Ashdod University Hospital Ashdod Israel; ^2^ Department of Interventional Radiology Samson Assuta Ashdod University Hospital Ashdod Israel

**Keywords:** complications, IVC injury, PCNL, vascular injury

## Abstract

This case underscores the importance of meticulous imaging and procedural vigilance during PCNL to prevent rare complications such as IVC penetration. A prompt multidisciplinary response and careful catheter repositioning ensured a favorable outcome, highlighting strategies to safely manage unexpected vascular injuries during urological procedures.

## 1 Introduction

PCNL is a minimally invasive surgical procedure utilized to remove large kidney stones that cannot be passed naturally or treated effectively with other methods. During PCNL, a small incision is made in the patient's back or flank, and access to the kidney's collecting system is achieved using an 8‐gauge needle, followed by tract dilation with a balloon or rigid dilators. A nephroscope is then inserted through an access sheath into the kidney to break up and extract the stone fragments. This procedure is typically performed under general anesthesia and guided by fluoroscopy or ultrasound to ensure precision.

PCNL is regarded as the gold standard for treating large renal stones [1] due to several advantages, including a high success rate in stone clearance, reduced pain and recovery time compared to open surgery, and the ability to manage stones that are too large or complex for other treatments. Common complications of PCNL include bleeding, infection, injury to surrounding organs, and residual stone fragments that may require further treatment. Bleeding complications are commonly arterial in nature. Here, we present a case of accidental penetration of the IVC during PCNL.

## 2 Case History

**FIGURE 1:**
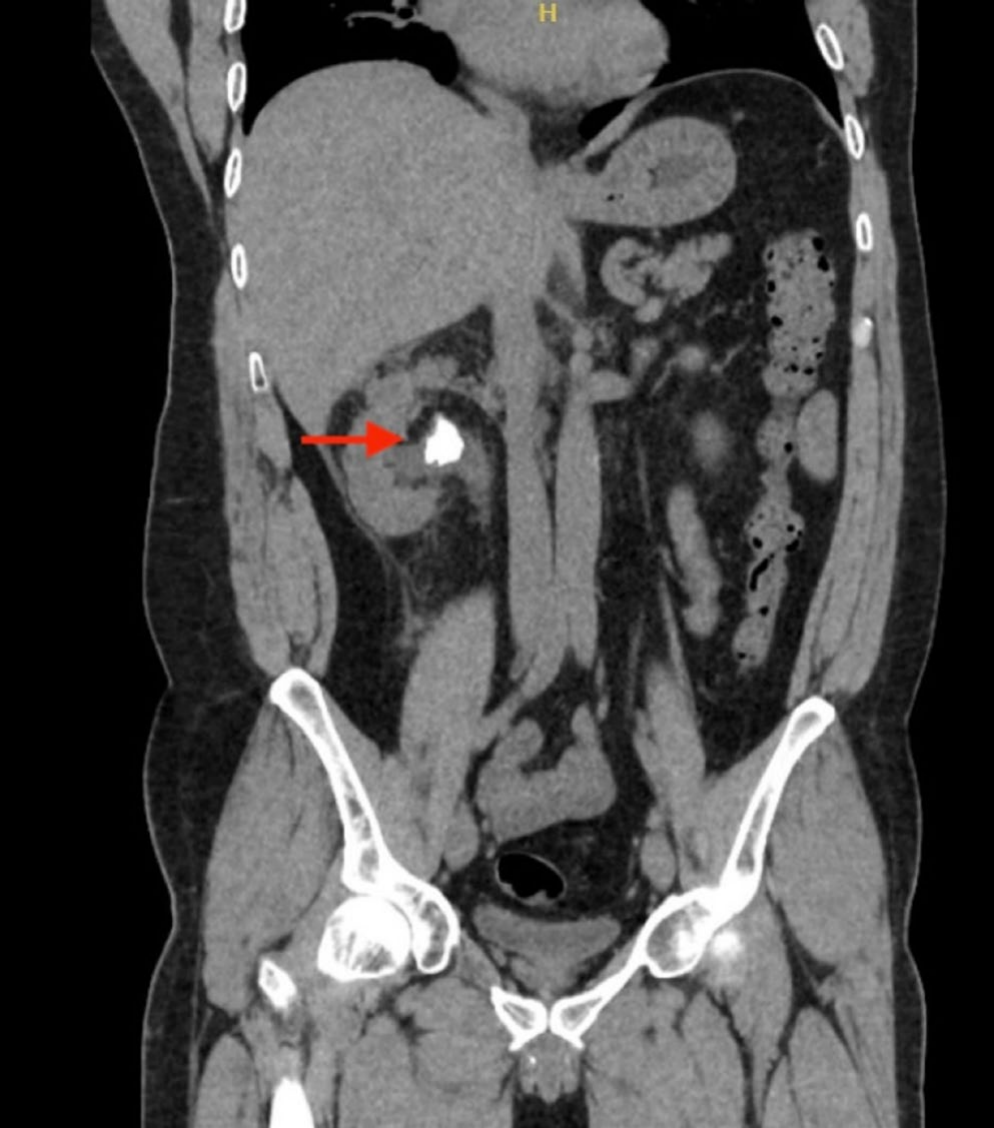
Coronal view of abdominal CT scan demonstrating a 2 cm calculus in the right renal pelvis.

**FIGURE 2:**
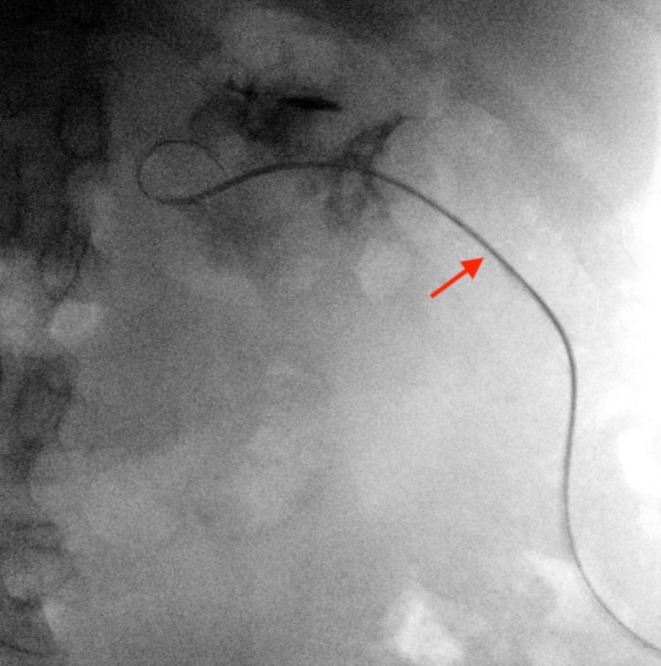
A Roadrunner hydrophilic wire guide passed through the nephrostomy tube into the renal pelvis.

**FIGURE 3:**
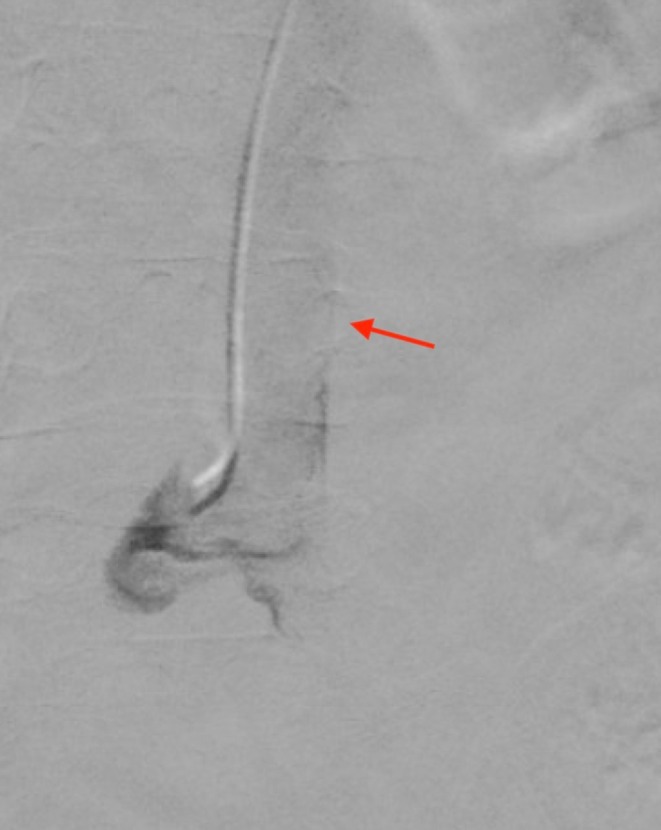
Fluoroscopic contrast injection through the Kumpe catheter revealing a wide tubular structure.

**FIGURE 4:**
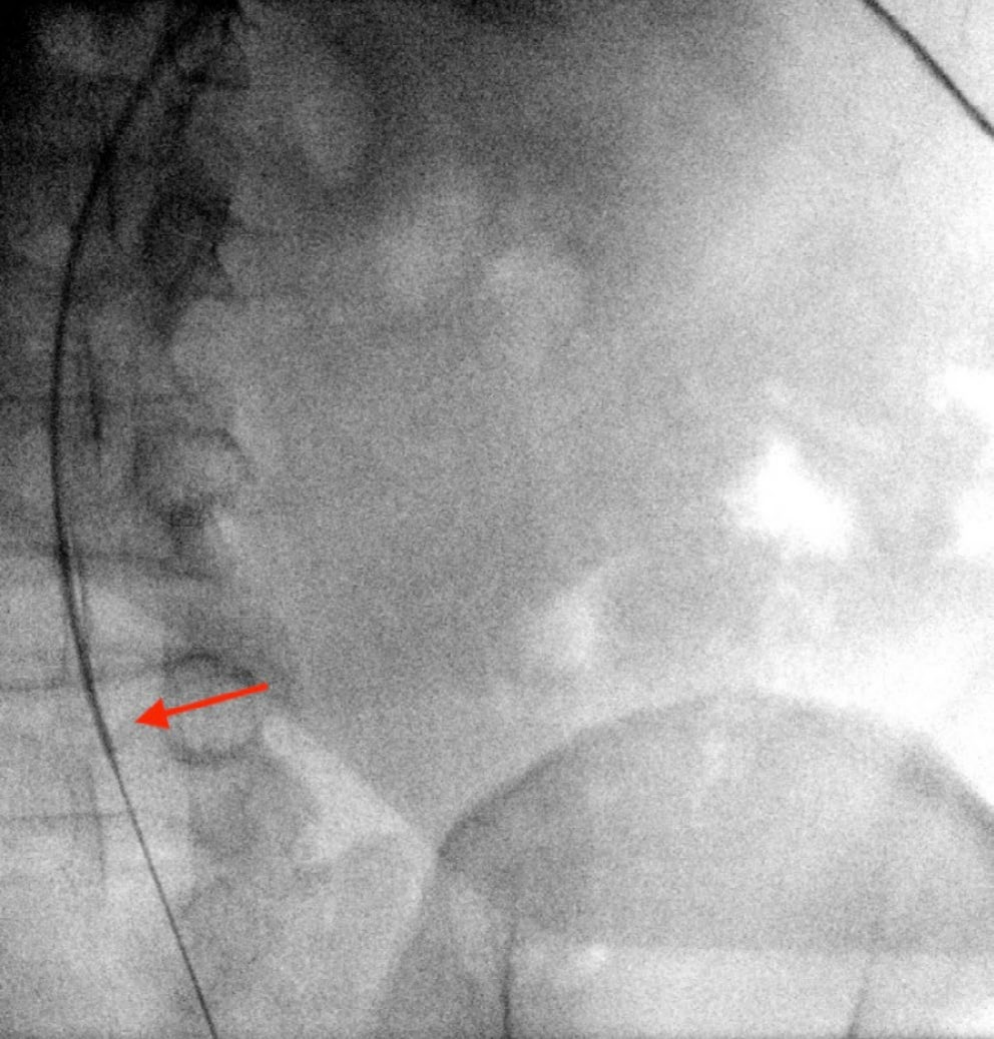
Kumpe catheter within the inferior vena cava.

A 43‐year‐old male was admitted to our department with right renal colic and fever. A non‐contrast CT scan revealed a large 2.5 cm obstructing stone in the renal pelvis (Figure 1). The patient received intravenous antibiotics, and an interventional radiologist inserted a NT into the middle calyx, which drained a purulent discharge. Three weeks later, an experienced endourologist performed a prone PCNL using the pre‐established NT tract. Initially, a Roadrunner PC Hydrophilic Wire Guide (Cook Medical) was advanced through the NT into the middle calyx in an attempt to pass the wire beyond the impacted stone (Figure 2). The NT was then removed. A Kumpe 5 Fr access catheter (Cook Medical) was then threaded over the wire, intended to guide it to the proximal ureter. Fluoroscopic contrast injection through the Kumpe catheter revealed a wide tubular structure, with rapid cranial wash‐out of the contrast material (Figure 3). Upon repeating the fluoroscopy with contrast‐enhanced material, it was concluded that the Kumpe catheter tip was located within the lumen of the IVC (Figure 4). An emergency multidisciplinary consultation was conducted, including an additional endourologist, vascular surgeon, interventional radiologist, and anesthesiologist. The patient was hemodynamically stable with no signs of active bleeding or increased respiratory pressure. The Kumpe catheter was carefully retracted with concurrent contrast injection, guiding it back into the middle calyx without bleeding through the tract into the renal unit or the retroperitoneum. The interventional radiologist then inserted an 8 Fr NT into the upper calyx via a new tract. The patient remained hemodynamically stable during the procedure. A subsequent CT with intravenous contrast showed no blush sign or evidence of bleeding. The patient later underwent successful PCNL from the upper pole to remove the stone.

## 3 Differential Diagnosis

This case underscores the importance of recognizing and managing rare complications during PCNL, such as inadvertent IVC catheterization. A swift multidisciplinary response, coupled with precise catheter guidance and imaging, is crucial to ensure favorable outcomes and minimize vascular injury during complex urological procedures.

## 4 Conclusion

Despite these risks, PCNL remains a highly effective treatment for large or complex kidney stones, offering a high success rate with relatively short recovery times compared to open surgery. Although rare, recognizing an inadvertent puncture of the IVC mandates a swift multidisciplinary response.

## 5 Discussion

This case report highlights the complexities and potential complications associated with PCNL, specifically the rare but significant event of accidental catheterization of the IVC. PCNL is a standard procedure for the removal of large renal stones, offering a minimally invasive alternative to open surgery with high success rates. However, it requires precise navigation and technique to avoid complications.

Sarwar et al. presented a case of renal vein injury during PCNL. They described that after tract dilatation and nephroscope insertion, they identified intense venous bleeding from the lower calyx. Following contrast injection, they identified the left renal vein. The bleeding was controlled using an Amplatz sheath over the bleeding tract, and a Double‐J stent was inserted [2].

Pan et al. reported on intensive hemorrhage following tract dilatation and access insertion during prone PCNL. The surgeon inserted a 16 Fr double lumen catheter into the working channel and dilated the balloon to compress the bleeding site. No bleeding was noted following the balloon dilatation, and the procedure was aborted. Postoperative CT scans demonstrated a thrombus in the IVC, which prompted insertion of a vascular filter and thrombus removal [3]. In our case, despite the fact that the NT was properly placed and drained urine properly, during the subsequent PCNL procedure, the Kumpe 5 Fr angled catheter, intended for the proximal ureter, was inadvertently advanced into the IVC. This misplacement was identified through contrast fluoroscopy, which revealed the unexpected path of the contrast material. We hypothesize that the pyonephrosis resulted in a fragile urothelium, prone to penetration, resulting in the wire easily transversing into the venous system. Such a complication, though rare, underscores the importance of careful imaging and verification of catheter placement during endourological procedures. Furthermore, the immediate multidisciplinary consultation and patient hemodynamic assessment was critical to the decision making, managing this complication. The Kumpe catheter was carefully removed from the IVC inorder to avoid fistula formation; at the same time, accessing the collecting system through a new tract to divert the urine from the injured calyx. The methodical retraction of the Kumpe catheter with concurrent contrast injection ensured that the catheter was safely relocated into the middle calyx without causing vascular injury or bleeding. The subsequent insertion of a new NT through a newly established tract reduced intrarenal pressure and allowed for monitoring of urine color to rule out delayed bleeding into the urinary system. This was confirmed by a follow‐up CT scan, which showed no signs of bleeding. Furthermore, the presence of the NT assisted in performing the RIRS with decreased intrarenal pressure, thereby avoiding potential complications. This case also demonstrates the value of a multidisciplinary approach and the need for prompt recognition and correction of procedural errors. The successful outcome was facilitated by the swift adaptation to the unexpected complication. This case has been reported in line with the SCARE criteria [4].

## Author Contributions

**Dor Golomb:** conceptualization, data curation, investigation, methodology, writing – original draft. **Michael Doberman:** writing – review and editing. **Sergey Litvin:** writing – review and editing. **Yuval Avda:** writing – review and editing. **Amir Cooper:** conceptualization, writing – review and editing. **Orit Raz:** conceptualization, writing – review and editing.

## Ethics Statement

This manuscript was exempt from ethical approval from the authors' institution.

## Consent

Written informed consent was obtained from the patient for the publication of this case report.

## Conflicts of Interest

The authors declare no conflicts of interest.

## Data Availability

The data that support the findings of this study are available on request from the corresponding author. The data are not publicly available due to privacy or ethical restrictions.
